# Ataxia Telangiectasia-Mutated (*ATM*)Polymorphisms and Risk of Lung Cancer in a Chinese Population

**DOI:** 10.3389/fpubh.2017.00102

**Published:** 2017-06-08

**Authors:** Ajay A. Myneni, Shen-Chih Chang, Rungui Niu, Li Liu, Baoxing Zhao, Jianping Shi, Xiaoyou Han, Jiawei Li, Jia Su, Shunzhang Yu, Zuo-Feng Zhang, Lina Mu

**Affiliations:** ^1^Department of Epidemiology and Environmental Health, School of Public Health and Health Professions, State University of New York (SUNY) at Buffalo, Buffalo, NY, United States; ^2^Department of Epidemiology, Fielding School of Public Health, University of California at Los Angeles (UCLA), Los Angeles, CA, United States; ^3^Shanxi Tumor Hospital, Taiyuan, Shanxi, China; ^4^Taiyuan City Center for Disease Control and Prevention (CDC), Taiyuan, Shanxi, China; ^5^School of Public Health, Fudan University, Shanghai, China

**Keywords:** lung cancer, ataxia telangiectasia-mutated gene, DNA repair, single-nucleotide polymorphisms, Chinese population

## Abstract

**Background:**

The ataxia telangiectasia-mutated (*ATM*) gene has a key role in DNA repair including activation and stabilization of *p53*, which implicates the importance of *ATM* polymorphisms in the development of cancer. This study aims to investigate the association of two *ATM* single-nucleotide polymorphisms (SNPs) with lung cancer, as well as their potential interaction with *p53* gene and other known risk factors of lung cancer.

**Methods:**

A population-based case–control study was conducted in Taiyuan city, China with 399 cases and 466 controls matched on the distribution of age and sex of cases. The two *ATM* gene SNPs, *ATM*rs227060 and *ATM*rs228589 as well as *p53* gene SNP, *p53*rs1042522 were genotyped using Sequenom platform. Unconditional logistic regression models were used to estimate crude and adjusted odds ratios (aOR) and 95% confidence intervals (CIs). Adjusted models controlled for age, sex, and smoking status.

**Results:**

The study showed that TT genotype of *ATM*rs227060 (aOR = 1.58, 95% CI: 1.06–2.35) and AA genotype of *ATM*rs228589 were significantly associated with lung cancer (aOR = 1.50, 95% CI: 1.08–2.08) in a recessive model. Additionally, carrying variant genotypes of *ATM*rs227060 (TT), *ATM*rs228589 (AA), and *p53*rs1042522 (CC) concomitantly was associated with much higher risk (aOR = 3.68, 95% CI: 1.43–9.45) of lung cancer than carrying variant genotypes of any one of the above three SNPs. We also found multiplicative and additive interaction between tea drinking and *ATM*rs227060 in association with lung cancer.

**Conclusion:**

This study indicates that *ATM* gene variants might be associated with development of lung cancer in Chinese population. These results need to be validated in larger and different population samples.

## Introduction

Lung cancer has been increasing both in incidence and mortality in China (1, 2). Smoking and air pollution are major risk factors of lung cancer in China. However, the disproportion between incidence of lung cancer and prevalence of smoking indicates that genetic factors may play an important role in the development of lung cancer in Chinese population (3).

The ataxia telangiectasia-mutated (*ATM)* gene is a Ser/Thr protein kinase of the phosphoinositidyl 3-kinase family located on long arm of chromosome 11q22–q23 (4). It is a key gene involved in DNA repair, mainly in double-strand breaks (5). It is rapidly recruited to the sites of DNA damage where it modulates downstream effectors [e.g., *p53, BRCA-1*, checkpoint kinase 2 (*CHK2*), *RAD50*, and *NBS1*] through phosphorylation of protein kinases and substrates. Cells with mutated *ATM* gene lose the ability to cope with genotoxic stresses due to lack of a coordinated DNA damage response (DDR) and loss of regulation of cell-cycle damage checkpoints (6). The principal component of *ATM*-mediated DDR involves the tumor-suppressor protein *p53*. *p53* gene regulates the transcription of other genes responsible for cellular antitumor responses such as cell-cycle arrest, apoptosis, senescence, genetic stability, and suppression of angiogenesis (7). Functional polymorphisms of *p53* gene alter protein activity and have been shown to be associated with risk of development of several human cancers including lung cancer (8–11). *ATM* mediates post-translational modifications of *p53* that lead to its activation and transcription of genes involved in cell-cycle arrest and apoptosis. In addition, modulation of proteins *CHK2* and MDM2 (which inhibits *p53* activation) results in rapid stabilization and accumulation of *p53* at the DDR sites (12). More recent studies suggest a wider role for *ATM* apart from involvement in DDR functions such as response to other forms of genotoxic stress like oxidative stress and maintaining cellular homeostasis including various cellular signaling pathways, insulin signaling, and mitochondrial homeostasis (6, 12, 13). This further indicates that polymorphisms of the *ATM* gene may be involved in cancer initiation and progression through multiple pathways.

Ataxia telangiectasia-mutated gene polymorphisms have been associated with several cancers including breast, oral, cervical, and lung (14–22). Among the *ATM* single-nucleotide polymorphisms (SNPs), we identified two intron variant tagging SNPs with *r*^2^ > 0.6 and minor allele frequency >5% (MAF), *ATM*rs227060 (MAF: 32.3%) and *ATM*rs228589 (MAF: 46.1%) (23–25). While these SNPs showed positive associations with other cancers, limited studies examined their association with lung cancer with inconsistent results. In previous studies, *ATM*rs227060 was positively associated with non-Hodgkin’s lymphoma while *ATM*rs228589 showed positive association with breast cancer in Ashkenazi Jewish women as well as chronic myeloid leukemia (26–28). In relation to lung cancer, a Caucasian study showed positive association of *ATM*rs227060 with non-small cell lung cancer (NSCLC) recent studies in Taiwanese population did not find an association of either SNP with lung cancer (15, 22). In the current study, we investigated the association of these two *ATM* SNPs with lung cancer in a Chinese population. In addition, we explored potential interaction between the *ATM* gene SNPs and the most studied *p53* gene SNP, *p53*rs1042522 in which, Arginine (Arg) is substituted by Proline (Pro) at codon 72 (Arg72Pro) (29). We also explored potential interaction between the *ATM* SNPs and smoking, alcohol intake, and tea drinking behaviors in association with lung cancer.

## Materials and Methods

### Study Population

A population-based case–control study was conducted in Taiyuan city, the capital of Shanxi Province, China. Incident cases were recruited between 2005 and 2007 from Shanxi Tumor Hospital, which catered to 70% of all cancer patients in Taiyuan city, Shanxi Province, China. Eligibility criteria for cases included age 20 years or older, residence in Taiyuan city for 10 years or more, medically stable condition and willingness to participate in the study. Controls were recruited from 13 randomly selected communities in Taiyuan to match cases according to distribution of age and gender. Eligible controls were aged 20 years or older, lived in Taiyuan city for 10 years or more and had no history of cancer or any other serious chronic disease. A total of 399 cases (89% response rate) and 466 controls (85% response rate) participated in the study. Detailed methodology for this population-based case–control study was previously published (30). Half of the participants were males and 56% of participants were aged 55 years or older. Around 47% of the participants were smokers, 25% of them reported alcohol intake, and 58% of them were current tea drinkers.

### Data Collection

Participants were interviewed face to face by study personnel using a structured questionnaire regarding their demographic characteristics, smoking, alcohol drinking and tea drinking habits, diet during the past 1 year, cooking and indoor heating methods, and individual and family medical history. Ever smokers were defined as those with a lifetime exposure of at least 100 cigarettes.

### Biospecimen Collection and Processing

Approximately 98% of cases and 99% of controls provided peripheral blood samples. Serum and blood clot were separated immediately and stored in a −80°C freezer. Genomic DNA was isolated for analysis using a modified phenol–chloroform method (31).

### Primer Design and Genotyping

Genotyping for *ATM* (rs227060 and rs228589) and *p53* (rs1042522) SNPs was performed using Sequenom platform (Sequenom, Inc., San Diego, CA, USA) in the Center for Genomics and Personalized Medicine Research at Wake Forest School of Medicine (Winston-Salem, NC, USA). Polymerase chain reaction (PCR) and extension primers were designed using MassARRAY Assay Design 3.1 software (Sequenom, Inc., San Diego, CA, USA). We followed the manufacturer’s iPLEX Application Guide (Sequenom, Inc., San Diego, CA, USA) in performing genotyping procedures. PCR reactions were carried out in 96-well plates. Two negative control (water) samples and two randomly selected replicated samples were randomly plated in each plate. The concordance rate (percentages of agreement) in quality control pairs was over 99%. Technicians were blinded to sample case/control status. The three SNPs conformed to Hardy–Weinberg equilibrium.

### Statistical Analysis

We used unconditional logistic regression models to analyze allele and genotype associations (in codominant, dominant, and recessive models) of the *ATM* SNPs with lung cancer by estimating crude and adjusted odds ratios (aOR) and 95% confidence intervals (CIs). In addition, we estimated crude and adjusted associations of risk genotype combinations of *ATM*rs227060, *ATM*rs228589, and *p53*rs1042522 with lung cancer. Adjusted models were controlled for age, sex, and smoking status (never smokers/ever smokers) of the participants. We performed internal validation for the genetic effect using a bootstrap method to obtain bias corrected estimates based on 1,000 replication data sets created by random sampling with replacement. Stratified analyses were used to assess the association of the SNPs with lung cancer among subgroups of age, sex, smoking, alcohol intake, tea drinking, and histological subtypes of lung cancer. For histological subtypes, the cases in each subgroup were compared to overall controls. In the current study, we assessed additive and multiplicative interaction of the each *ATM* SNP with *p53* SNP (gene–gene) as well as with other risk factors (gene–environment) like smoking, alcohol drinking, and tea drinking exposures. Multiplicative interaction was assessed by introducing a product term in logistic regression models. Additive interaction was assessed by estimating relative excess risk due to interaction (RERI). All analyses were performed using SAS 9.3 software. Microsoft Excel was used additionally for estimating RERI.

## Results

The description of sociodemographic and behavioral characteristics of cases and controls has been presented in detail in a previous publication (30). Briefly, a higher proportion of cases were heavy smokers and tea non-drinkers. Education level, BMI, and average household annual income 10 years prior were all higher among controls. Analysis of association of the *ATM* SNPs with lung cancer has been shown in Table 1. The frequency of C and T alleles of *ATM*rs227060 was 64.2 and 35.8% in controls and the frequency of T and A alleles *ATM*rs228589 was 53.1 and 46.9% in controls, respectively. Lung cancer was significantly associated with TT genotype of *ATM*rs227060 in a recessive model (aOR = 1.58, 95% CI: 1.06–2.35). AA genotype of *ATM*rs228589 was also significantly associated with lung cancer in a recessive model (aOR = 1.50, 95% CI: 1.08–2.08). Among the haplotypes identified from *ATM*rs227060 and *ATM*rs228589 (data not shown), TTAA haplotype was more frequent among cases compared to controls but failed to show an association with lung cancer (aOR = 1.53, 95% CI: 0.95–1.47). Internal validation using a bootstrap procedure with 1,000 replicated data sets confirmed the statistically significant association estimates between the *ATM* SNPs and lung cancer (results not shown).

**Table 1:** **Association of *ATM* gene polymorphisms with lung cancer**.

Gene	Genotype	Cases *N* (%)	Controls *N* (%)	Crude odds ratio (OR) (95% CI)	Adjusted odds ratios^a^ (95% CI)
*ATM* (rs227060)		352	451		
	CC	137 (38.9)	183 (40.6)	1.00	1.00
	CT	150 (42.6)	213 (47.2)	0.94 (0.69–1.28)	0.93 (0.68–1.28)
	TT	65 (18.5)	55 (12.2)	**1.58 (1.04–2.41)**	1.52 (0.99–2.35)
Dominant model	CT + TT	215 (61.1)	268 (59.4)	1.07 (0.81–1.43)	1.06 (0.79–1.42)
Recessive model (ref: CC + CT)	TT	65 (18.5)	55 (12.2)	**1.63 (1.10–2.41)**	**1.58 (1.06–2.35)**
Allele OR	T			1.18 (0.96–1.44)	1.16 (0.94–1.42)
*ATM* (rs228589)		360	448		
	TT	103 (28.6)	127 (28.4)	1.00	1.00
	TA	150 (41.7)	222 (49.6)	0.83 (0.60–1.16)	0.81 (0.57–1.14)
	AA	107 (29.7)	99 (22.0)	1.33 (0.91–1.94)	1.31 (0.89–1.94)
Dominant model	TA + AA	257 (71.4)	321 (71.7)	0.99 (0.73–1.34)	0.96 (0.70–1.32)
Recessive model (ref: TT + TA)	AA	107 (29.7)	99 (22.0)	**1.49 (1.09–2.05)**	**1.50 (1.08–2.08)**
Allele OR	A			1.15 (0.95–1.39)	1.14 (0.94–1.38)

*Estimates in bold represent statistically significant associations*.

*^a^Adjusted for age, sex, and smoking status (0 = never smokers, 1 = ever smokers)*.

**Figure 1:**
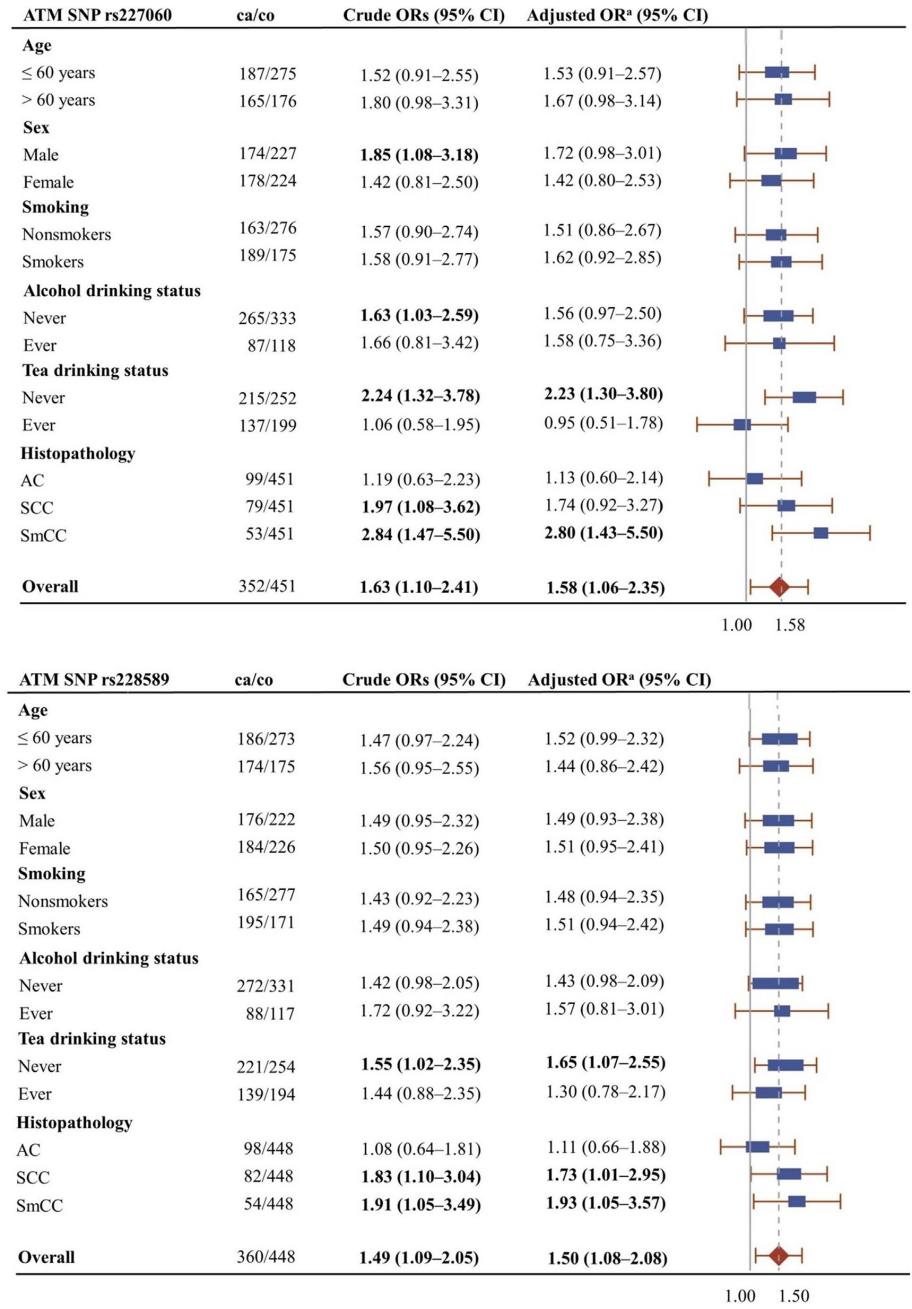
**Forest plots showing the association of *ATM*rs227060 and *ATM*rs228589 with lung cancer among different subgroups**. Plot shows adjusted odds ratios. ^a^Adjusted for age, sex, and smoking status (0 = never smokers, 1 = ever smokers). ca/co, number of cases/controls; AC, adenocarcinoma; SCC, squamous cell carcinoma; SmCC, small cell carcinoma. The solid line in the plot represents null value and the broken line represents overall odds ratio (OR). Size of the square represents the inverse of the variance of the log OR. ORs in bold represent statistically significant associations.

Figure 1 shows the association of *ATM*rs227060 and *ATM*rs228589 with lung cancer among risk factor subgroups. *ATM*rs227060 (aOR = 2.23, 95% CI: 1.30–3.80) and *ATM*rs228589 (aOR = 1.65, 95% CI: 1.07–2.55) showed a significant association with lung cancer among tea non-drinkers. Among histological subtypes of lung cancer, *ATM*rs227060 was significantly associated with small cell carcinoma (SmCC) while *ATM*rs228589 was significantly associated with squamous cell carcinoma (SCC) and SmCC.

A previous publication reported the association of *p53*rs1042522 SNP with lung cancer (32). Since the role of *ATM* gene in DNA repair closely relates to the function of *p53* gene, in the current study, we analyzed the association of lung cancer with different genotype combinations of *ATM*rs227060, *ATM*rs228589, and *p53*rs1042522. The results are presented in Table 2, which shows a statistically significant association with lung cancer in presence of risk genotypes of all three SNPs (aOR = 3.68, 95% CI = 1.43–9.45). Test for linear trend showed statistical significance (*p*_trend_ = 0.002). Similar to the results in Table 1, internal validation using the bootstrap procedure described before confirmed the statistically significant associations in Table 2 (results not shown).

**Table 2:** **Association of combined genotypes of *ATM*rs227060, *ATM*rs228589, and *p53*rs1042522 with lung cancer**.

No. of risk genotypes	*ATM*rs227060	*ATM*rs228589	*p53*rs1042522	Cases *N* (%)	Controls *N* (%)	Crude odds ratio (95% CI)	Adjusted odds ratios^a^ (95%CI)
				333	441		
0	CC + CT	TT + TA	GG + GC	173 (52.0)	272 (61.7)	1.00	1.00
1	CC + CT	AA	GG + GC	92 (27.6)	108 (24.5)	1.34 (0.96–1.88)	1.39 (0.98–1.96)
	CC + CT	TT + TA	CC				
	TT	TT + TA	GG + GC				
2	CC + CT	AA	CC	53 (15.9)	54 (12.2)	**1.54 (1.01–2.36)**	1.49 (0.97–2.31)
	TT	AA	GG + GC				
	TT	TT + TA	CC				
3	TT	AA	CC	15 (4.5)	7 (1.6)	**3.37 (1.35–8.43)**	**3.68 (1.43–9.45)**
	*p* for trend					**0.002**	**0.002**

*Estimates in bold represent statistically significant associations; p values in bold represent statistically significant linear trend*.

*^a^Adjusted for age, sex, and smoking status (0 = never smokers, 1 = ever smokers)*.

Based on the results from Table 2, we tested for gene–gene interaction between each of the *ATM* SNPs and the *p53* SNP. We did not find statistically significant multiplicative or additive interaction between *ATM*rs227060–*p53*1042522 and *ATM*rs228589–*p53*1042522 (Table 3). We observed statistically significant mul-tiplicative and additive interaction between *ATM*rs227060 and tea drinking but not smoking and alcohol drinking in association with lung cancer. Smoking, alcohol drinking, or tea drinking did not modify the association between *ATM*rs228589 and lung cancer (Table 3).

**Table 3:** **Potential gene–gene and gene–environment interactions of *ATM* gene polymorphisms in association with lung cancer**.

		Cases *N* (%)	Controls *N* (%)	Crude odds ratio (OR) (95% CI)	Adjusted odds ratios^a^ (95% CI)
***p53*rs1042522**	***ATM*rs227060**				
GG + GC	CC + CT	205 (60.1)	310 (70.0)	1.00	1.00
GG + GC	TT	44 (12.9)	45 (10.2)	1.48 (0.94 to 2.32)	1.38 (0.87 to 2.19)
CC	CC + CT	75 (22.0)	80 (18.0)	1.42 (0.99 to 2.03)	**1.44 (1.00 to 2.10)**
CC	TT	17 (5.0)	8 (1.8)	**3.21 (1.36 to 7.58)**	**3.36 (1.39 to 8.08)**
OR for interaction^b^				1.53 (0.56 to 4.19)	1.68 (0.60 to 4.71)
RERI^c^				1.32 (−1.49 to 4.12)	1.53 (−1.45 to 4.51)
**Smoking**					
Never	CC + CT	136 (38.6)	245 (54.3)	1.00	1.00
Never	TT	27 (7.7)	31 (6.9)	1.57 (0.90 to 2.74)	1.52 (0.87 to 2.68)
Ever	CC + CT	151 (42.9)	151 (33.5)	**1.80 (1.32 to 2.45)**	**3.69 (2.30 to 5.92)**
Ever	TT	38 (10.8)	24 (5.3)	**2.85 (1.64 to 4.96)**	**6.05 (3.10 to 11.84)**
OR for interaction				1.01 (0.46 to 2.22)	1.08 (0.48 to 2.40)
RERI				0.48 (−1.24 to 2.20)	1.84 (−1.63 to 5.32)
**Alcohol drinking**					
Never	CC + CT	219 (62.2)	295 (65.4)	1.00	1.00
Never	TT	46 (13.1)	38 (8.4)	1.63 (1.03 to 2.59)	1.56 (0.97 to 2.51)
Ever	CC + CT	68 (19.3)	101 (22.4)	0.91 (0.64 to 1.29)	0.71 (0.47 to 109)
Ever	TT	19 (5.4)	17 (3.8)	1.51 (0.77 to 2.96)	1.21 (0.58 to 2.51)
OR for interaction				1.02 (0.43 to 2.40)	1.08 (0.45 to 2.61)
RERI				−0.03 (−1.29 to 1.22)	−0.07 (−1.17 to 1.04)
**Tea drinking**					
Ever	CC + CT	116 (32.9)	170 (37.7)	1.00	1.00
Ever	TT	21 (6.0)	29 (6.4)	0.89 (0.43 to 1.87)	0.96 (0.51 to 1.79)
Never	CC + CT	171 (48.6)	226 (50.1)	1.37 (0.92 to 2.05)	1.29 (0.90 to 1.86)
Never	TT	44 (12.5)	26 (5.8)	**2.43 (1.31 to 4.53)**	**2.89 (1.63 to 5.12)**
OR for interaction				2.11 (0.94 to 4.70)	**2.32 (1.02 to 5.29)**
RERI				1.31 (−0.07 to 2.69)	**1.63 (0.05 to 3.21)**

***p53*rs1042522**	***ATM*rs228589**				
GG + GC	TT + TA	180 (51.9)	274 (62.0)	1.00	1.00
GG + GC	AA	73 (21.0)	81 (18.3)	1.37 (0.95 to 1.98)	1.35 (0.92 to 1.97)
CC	TT + TA	65 (18.7)	71 (16.1)	1.39 (0.95 to 2.05)	1.42 (0.95 to 2.11)
CC	AA	29 (8.4)	16 (3.6)	**2.76 (1.46 to 5.23)**	**3.00 (1.55 to 5.83)**
OR for interaction				1.44 (0.66 to 3.17)	1.57 (0.70 to 3.55)
RERI				0.99 (−0.81 to 2.80)	1.24 (−0.78 to 3.25)
**Smoking**					
Never	TT + TA	119 (33.1)	218 (48.7)	1.00	1.00
Never	AA	46 (12.8)	59 (13.2)	1.43 (0.92 to 2.23)	1.48 (0.94 to 2.33)
Ever	TT + TA	134 (37.2)	131 (29.2)	**1.87 (1.35 to 2.60)**	**4.14 (2.51 to 6.83)**
Ever	AA	61 (16.9)	40 (8.9)	**2.79 (1.77 to 4.41)**	**6.29 (3.45 to 11.48)**
OR for interaction				1.04 (0.55 to 1.99)	1.03 (0.53 to 1.98)
RERI				0.49 (−1.04 to 2.02)	1.67 (−1.27 to 4.61)
**Alcohol drinking**					
Never	TT + TA	193 (53.6)	257 (57.4)	1.00	1.00
Never	AA	79 (21.9)	74 (16.5)	1.42 (0.98 to 2.05)	1.42 (0.97 to 2.09)
Ever	TT + TA	60 (16.7)	92 (20.5)	0.87 (0.60 to 1.26)	0.69 (0.44 to 1.08)
Ever	AA	28 (7.8)	25 (5.6)	1.49 (0.84 to 2.64)	1.17 (0.62 to 2.21)
OR for interaction				1.21 (0.58 to 2.51)	1.20 (0.57 to 3.53)
RERI				0.20 (−0.77 to 1.18)	0.06 (−0.80 to 0.92)
**Tea drinking**					
Ever	TT + TA	116 (32.9)	170 (37.7)	1.00	1.00
Ever	AA	21 (6.0)	29 (6.4)	1.44 (0.88 to 2.35)	1.31 (0.79 to 2.17)
Never	TT + TA	171 (48.6)	226 (50.1)	1.20 (0.87 to 1.68)	1.37 (0.93 to 2.00)
Never	AA	44 (12.5)	26 (5.8)	**1.86 (1.19 to 2.91)**	**2.27 (1.39 to 3.70)**
OR for interaction				1.07 (0.56 to 2.04)	1.27 (0.65 to 2.47)
RERI				0.22 (−0.74 to 1.18)	0.59 (−0.49 to 1.68)

*Estimates in bold represent statistically significant associations*.

*^a^Adjusted for age, sex, and smoking status (0 = never smokers, 1 = ever smokers)*.

*^b^Results of multiplicative interaction*.

*^c^Results of relative excess risk due to interaction. RERI value “0” indicates no biological interaction*.

## Discussion

In the current study, *ATM*rs227060 (TT) and *ATM*rs228589 (AA) were positively associated with lung cancer in recessive models. In addition, both showed positive association with lung cancer in tea non-drinkers and with SmCC subtype. Bonnen et al. earlier reported high linkage disequilibrium (LD) in the ATM gene locus (33). We verified this information from publicly available databases (HapMap and 1000 Genomes Project) for *ATM*rs227060 and *ATM*rs228589 (*r*^2^ range: 61.9–75.2) (23, 24) in Chinese population samples. This may explain the similar associations of the two *ATM* SNPs with lung cancer in our study. Both these SNPs may have a role in modifying the *ATM* gene function and consequently altering DNA repair mechanisms. A previous study suggested that *ATM*rs227060 may potentially regulate PI3 kinase activity because of its proximity to the *ATM* PI3 kinase domain (22). Another study showed that *ATM*rs228589 modulates DNA repair capacity of UV damaged DNA, which may be due to defects in *ATM* signaling (34). However, it is also possible that these tagging SNPs may not be the causal polymorphisms but merely represent other functional polymorphisms that are in high LD with the current SNPs [e.g., rs189037 (35, 36), rs652311 (15), rs170548 (22), rs664143 (16)].

In the presence of *p53*rs1042522 variant genotype (CC) in addition to variant genotypes of *ATM*rs227060 (TT) and *ATM*rs228589 (AA), the aOR for developing lung cancer was 3.68 (95% CI: 1.43–9.45), which was 2.65 times higher than with the presence of variant genotype of any one of the three SNPs (aOR = 1.39, 95% CI: 0.98–1.96). Analysis for linear trend indicated significant gene-dosage effects of the risk genotypes of the three SNPs in association with lung cancer. This suggests that the risk of development of lung cancer associated with polymorphisms in *ATM* gene variants is much higher in a presence of coexisting mutations in the *p53* gene.

A recent case–control study in Taiwanese population (358 cases and 716 controls) examined the association of *ATM* polymorphisms, including *ATM*rs227060 and *ATM*rs228589 with lung cancer. The distribution of variant genotypes among controls for both the *ATM* SNPs [*ATM*rs227060 (TT: 14.3%) and *ATM*rs228589 (AA: 21.0%)] was similar to the controls in the current study [*ATM*rs227060 (TT: 12.2%) and *ATM*rs228589 (AA: 22.0%)]. However, the Taiwanese study did not find a difference in proportion of the variant genotypes of either *ATM*rs227060 or *ATM*rs228589 between lung cancer cases and healthy controls (15). We are uncertain whether differences in participant selection or other additional factors contributed to the variation in findings between the two studies. Another study in a Caucasian population investigated the association of *ATM* SNPs with NSCLC (556 cases and 556 controls). The allele frequencies of the variant genotypes of the two *ATM* SNPs in the Caucasian study were different from the current study [*ATM*rs227060 (TT: 7.6%) and *ATM*rs228589 (AA: 31.5%)]. The Caucasian study reported a significant association between *ATM*rs227060 (C>T) and lung cancer (OR = 1.55, 95% CI: 1.02–2.35) after controlling for age, gender, and smoking status, which was similar to findings in the current study for *ATM*rs227060 (C>T) (OR = 1.58, 95% CI: 1.06–2.35). No significant association was reported for *ATM*rs228589 in association with NSCLC in the Caucasian study (22). All three existing studies including the current study that investigated the association of the *ATM* SNPs (rs227060 and rs228589) and lung cancer had relatively small sample size. This may have contributed to the inconsistency in findings between the studies, requiring further evaluation of these associations in larger and different populations.

In the stratified analysis, the association between *ATM*rs227060 or *ATM*rs228589 and lung cancer in our study population was similar among smokers and non-smokers as evidenced by the slightly elevated ORs of similar magnitude in these subgroups. These results were different from the Caucasian study, which reported a significant association of *ATM*rs227060 only among former smokers (22). In the current study, *ATM*rs227060 was significantly associated with lung cancer among tea non-drinkers (aOR = 2.23, 95% CI: 1.30–3.80) but not among tea ever drinkers (aOR = 0.95, 95% CI: 0.51–1.78). Tea is a popular beverage in China. In our study population, 42% of participants currently/formerly consumed tea. Tea drinking was found to be significantly protective against lung cancer in current drinkers (aOR = 0.54, 95% CI: 0.37–0.80; results not shown) in our study population. The flavins and catechins—mainly epigallocatechin gallate—in tea, especially green tea, contribute to its antitumor properties such as inhibiting oxidative damage, modulating cell signaling pathways to prevent tumorigenesis, and promoting apoptosis and cell-cycle arrest in cancer cells (37). In our exploratory interaction analysis, we observed statistically significant multiplicative and additive interaction between *ATM*rs227060 and tea drinking in association with lung cancer where carrying the variant genotype *ATM*rs227060TT and not consuming tea was associated with a higher risk of lung cancer compared to carrying *ATM*rs227060TT or not drinking tea alone. These results suggest that the association between *ATM* SNPs and lung cancer may be modified by tea drinking. However, given the small sample size among the tea drinking subgroups, the results we observed might be due to chance. When stratified by histological type of lung cancer, we observed that both *ATM*rs227060 and *ATM*rs228589 were significantly associated with SCC (a subtype of NSCLC) and SmCC. We did not find statistically significant additive or multiplicative interaction between the either of the *ATM* SNPs and smoking or alcohol drinking factors.

Ataxia telangiectasia-mutated gene serves as a crucial player in DDR mainly by regulating the stabilization and activation of *p53* (12, 38). Activated *p53* modulates the expression of genes involved in either activation of cell-cycle checkpoints or apoptosis (39). Cell-cycle arrest provides time to repair damaged DNA and if the damage is irreparable, *p53* promotes apoptosis (12). Mutations in the *ATM* gene could disrupt this finely regulated process and cause persistent and unrepaired DNA damage. This damage leads to chromosomal aberrations, which increase the risk of cancer development. *In vitro* studies have reported the importance of *ATM*-mediated phosphorylation in IR-induced checkpoint activation (40). Previous studies reported higher genetic instability and lower *ATM* protein expression in lung cancer patients compared to controls (41). Our results support the experimental study findings on the role of *ATM* SNPs in development of lung cancer especially in individuals with homozygous variant alleles.

A limitation of our study is the relatively small sample size. In particular, the association of *ATM* haplotypes and *ATM* SNPs with lung cancer among risk factor strata may have been affected by the smaller subgroup samples and these results should be interpreted cautiously. However, the associations that we found in our study contribute to the understanding of the potential role of *ATM* gene polymorphisms in the development of lung cancer and justify the need to pursue validation in larger studies.

In conclusion, our results indicate that having variant genotypes of *ATM*rs227060 (C>T) and *ATM*rs228589 (T>A) are independently associated with increased risk of developing lung cancer in a Chinese population. Further, carriers of variant genotypes of *ATM*rs227060, *ATM*rs228589, and *p53*rs1042522 (G>C) have a significantly increased risk for lung cancer compared to carriers of variant genotypes of the SNPs individually.

## Ethics Statement

Before initiation of participant recruitment, IRB approvals were obtained from Fudan University (IRB#04-10-0022) and UCLA (IRB#11-003153), respectively. A written informed consent was obtained from each participant.

## Author Contributions

LM: design and implementation of the study as well as supervision of analysis and manuscript preparation. AM: data analysis and manuscript preparation. S-CC: sample processing, genotyping, and editing the manuscript. RN, LL, BZ, JS, XH, LM: participant recruitment and data collection. JL, JS, and LL: monitoring data collection and study implementation. SY: study implementation. Z-FZ: study design and implementation.

## Conflict of Interest Statement

The authors declare that this research was conducted in the absence of any commercial or financial relationships that could be construed as a potential conflict of interest.
